# Longipin: An Amyloid Antimicrobial Peptide from the Harvestman *Acutisoma longipes* (Arachnida: Opiliones) with Preferential Affinity for Anionic Vesicles

**DOI:** 10.1371/journal.pone.0167953

**Published:** 2016-12-20

**Authors:** Raphael Santa Rosa Sayegh, Isabel de Fátima Correia Batista, Robson Lopes de Melo, Karin A. Riske, Sirlei Daffre, Guillermo Montich, Pedro Ismael da Silva Junior

**Affiliations:** 1 Programa Interunidades em Biotecnologia, Instituto de Ciências Biomédicas, Universidade de São Paulo, São Paulo, Brazil; 2 Laboratório Especial de Toxinologia Aplicada, Instituto Butantan, São Paulo, Brazil; 3 Unidade de Sequenciamento de Proteínas e Peptídeos, Instituto Butantan, São Paulo, Brazil; 4 Laboratório de Bioquímica e Biofísica, Instituto Butantan, São Paulo, Brazil; 5 Departamento de Biofísica, Universidade Federal de São Paulo, São Paulo, Brazil; 6 Departamento de Parasitologia, Instituto de Ciências Biomédicas, Universidade de São Paulo, São Paulo, Brazil; 7 Centro de Investigaciones en Quimica Biológica de Córdoba (CIQUIBIC, UNC-CONICET), Departamento de Química Biológica, Facultad de Ciencias Químicas, Universidad Nacional de Córdoba, Córdoba, Argentina; Bose Institute, INDIA

## Abstract

In contrast to vertebrate immune systems, invertebrates lack an adaptive response and rely solely on innate immunity in which antimicrobial peptides (AMPs) play an essential role. Most of them are membrane active molecules that are typically unstructured in solution and adopt secondary/tertiary structures upon binding to phospholipid bilayers. This work presents the first characterization of a constitutive AMP from the hemolymph of an Opiliones order animal: the harvestman *Acutisoma longipes*. This peptide was named longipin. It presents 18 aminoacid residues (SGYLPGKEYVYKYKGKVF) and a positive net charge at neutral pH. No similarity with other AMPs was observed. However, high sequence similarity with heme-lipoproteins from ticks suggested that longipin might be a protein fragment. The synthetic peptide showed enhanced antifungal activity against *Candida guilliermondii* and *C*. *tropicalis* yeasts (MIC: 3.8–7.5 μM) and did not interfered with VERO cells line viability at all concentrations tested (200–0.1 μM). This selectivity against microbial cells is related to the highest affinity of longipin for anionic charged vesicles (POPG:POPC) compared to zwitterionic ones (POPC), once microbial plasma membrane are generally more negatively charged compared to mammalian cells membrane. Dye leakage from carboxyfluorescein-loaded POPG:POPC vesicles suggested that longipin is a membrane active antimicrobial peptide and FT-IR spectroscopy showed that the peptide chain is mainly unstructured in solution or in the presence of POPC vesicles. However, upon binding to POPG:POPC vesicles, the FT-IR spectrum showed bands related to β-sheet and amyloid-like fibril conformations in agreement with thioflavin-T binding assays, indicating that longipin is an amyloid antimicrobial peptide.

## 1 Introduction

The invertebrate immune system lacks a specific response against invading microorganisms because it is unable to produce antibodies that can specifically recognize these pathogens. Different from the vertebrates, which possess an adaptive response, their defense mechanisms against invading agents rely solely on the innate immunity [1].

Cellular and humoral reactions are part of invertebrate innate immunity and act in concert to combat invading agents. Phagocytosis, which is performed by hemocytes, is part of the cellular response and can directly eliminate microorganisms. The clotting cascade and the action of antimicrobial peptides (AMPs) are part of the humoral response. In addition to avoiding hemolymph leakage, clotting can physically trap microorganisms, thereby favoring the action of antimicrobial molecules such as AMPs that are involved in direct microbial killing [2, 3]. Therefore, AMPs play an important role in invertebrate innate immunity.

The expression of AMPs in invertebrates can be either constitutive or induced after microbial challenge [1]. An inducible mechanism is thought to have appeared later in evolution due to the complexity of the microbial recognition machinery [4]. This observation is in agreement with several works that have shown the presence of constitutive AMPs in primitive invertebrate groups (Mollusca [4], Merostomata [5] Aranae [6–8], Scorpiones [9], and Acari [10]) and inducible AMPs in holometabolous insects [11], which is a higher invertebrate group. These works also suggest that the constitutive expression of AMPs is a synapormophy of class Chelicerata, to which order Opiliones belongs. Thus, there is interest in characterizing AMPs in different invertebrate groups to understand their evolutionary history.

AMPs are amphipathic molecules that generally present a positive net charge at neutral pH and have sizes ranging from 9 to 100 residues [12, 13] that have been purified from virtually every form of life [14]. They can be gene-encoded molecules, such as gomesin that is expressed as a pro-peptide [15], or originate from protein cleavage, such as has been observed in shrimp [16], ticks [17] and spiders [18]. These peptides can be grouped according to their physicochemical and structural characteristics [19] as well as their spectrum of activity.

Most AMPs are unstructured in solution but adopt secondary/tertiary structure elements when bound to phospholipid membranes. This feature is closely related to their usual mode of action: most AMPs are membrane active molecules. They can disrupt the phospholipid bilayer by mechanisms that include the formation of transient pores that can cause the loss of intracellular content and lead to microorganism death [12, 20]. Proposed mechanisms of action of membrane active AMPs include: (i) formation of a “toroidal” pore, where an aggregate of peptide chains inserts into the membrane and forms a pore with an internal region composed of phospholipid polar heads and the hydrophilic side chains of the peptide; (ii) a “barrel stave” pore, where the internal region of the pore is composed only of hydrophilic side chains; (iii) the carpet mechanism, where a complete disruption of the membrane occurs in a detergent-like manner [20] and (iv) the “leaky slit” mode, where the peptides are inserted perpendicularly into the membrane in an amyloid-like fiber conformation with the hydrophobic residues facing the phospholipid tails and the hydrophilic residues forcing lipids to adopt a positive curvature to form a slit [21].

Recently, the antimicrobial activity of amyloid peptides has been reviewed [22], and the existence of a new class of membrane active AMPs has been suggested. These peptides can adopt β-like structures similar to Alzheimer disease’s β-amyloid protein upon biding to membranes. Jang et al. [23] showed by atomic force microscopy and molecular dynamics simulation that protegrin-1 (an antimicrobial peptide found in human leukocytes) small oligomers could form channels in phospholipid membranes that were very similar to Alzheimer’s β-amyloid channels. Thus, these works point to the relationship between the pathogenesis of amyloid diseases and the mode of action of antimicrobial peptides.

The appearance of AMP resistance is considered unlikely due to their mode of action. For this reason, the development of new AMP-based drugs for the treatment of infectious diseases has become an area of intense research [24]. However, several microbial strategies related to AMP resistance have appeared [25] and should be considered for the development of new antimicrobial drugs.

This is the first report on the purification, characterization and binding to lipid membranes of an antimicrobial peptide from the hemolymph of an Opiliones order animal: the harvestman *Acutisoma longipes* [26]. We called this peptide longipin. Here, we show that it adopts an amyloid-like fibril conformation upon binding to anionic vesicles and increases membrane permeability. In addition to allowing the discovery of new molecules that have an alternative mode of action compared to conventional antibiotics, the study of AMPs from different groups of animals contributes to a wider understanding of the immunological system and the origin of its mechanisms in evolutionary history.

## 2 Material and Methods

### 2.1 Animal Capture and Hemolymph Extraction

*Acutisoma longipes* harvestmen of both genders and at different stages of development were collected in grottoes near “Pedra Gande” in Atibaia city (São Paulo, Brazil, 23°10′10.8″S 46°31′40.0″W) under an IBAMA license (n° 11024–3). The animals were kept under aseptic conditions for at least 10 days prior to hemolymph extraction.

Hemolymph was extracted from pre-chilled animals (-20°C for 3 min) by puncturing the fourth leg via coxae-trocanter articulation with an apyrogenic syringe. To avoid hemolymph coagulation, the extraction was performed in the presence of sodium citrate buffer (0.45 M NaCl, 0.1 M glucose, 30 mM trisodium citrate, 26 mM citric acid, and 10 mM EDTA; pH 4.2) [6]. The total volume of hemolymph used in this work (1.5 ml) was extracted from approximately 150 animals.

### 2.2 Hemolymph Fractionation

Hemolymph extracted from unchallenged harvestmen in the presence of sodium citrate buffer was centrifuged (800 × g for 10 min, 4°C) and divided into its two major components: cell-free plasma and hemocytes.

Plasma was resuspended in 15 ml of 0.05% trifluoroacetic acid (TFA), stirred on ice for 30 min, and centrifuged (16,000 × g for 30 min, 4°C). The supernatant was concentrated in a vacuum centrifuge and resuspended in 5 ml of 0.05% TFA. This plasma extract was directly applied into three Sep-Pak^®^ Plus tC18 cartridges (Waters, USA) equilibrated with 0.05% TFA. Three stepwise elutions with 5%, 40% and 80% ACN in 0.05% TFA were performed to fractionate the plasma.

The material eluted with 40% ACN from the Sep-Pak was concentrated in a vacuum centrifuge, resuspended in 1 ml of 0.05% TFA and subjected to the first purification step by RP-HPLC with a semi-preparative Vydac^®^ (Grace, USA) C18 column (5 μm, 250 × 10 mm). The elution was performed in a 2–60% gradient of ACN/0.05% TFA in H_2_O/0.05% TFA over 120 min with a 1.5 ml/min flow rate. The absorbance was monitored at 225 nm on a UFLC Prominence device (Shimadzu, Japan). The resultant fractions were collected manually and used in the antimicrobial activity assay.

Active fractions from the first step were subjected to a second purification step by RP-HPLC with a Vydac^®^ C18 (5 μm, 250 × 4.6 mm) analytical column. Optimized ACN/0.05% TFA gradients in H_2_O/0.05% TFA were used over 60 min with a 1 ml/min flow rate. All fractions were collected manually and evaluated in the microbial growth inhibition assay.

### 2.3 Microorganisms

*Escherichia coli* SBS 363 and *Micrococcus luteus* A270 were obtained from the Pasteur Institute collection [6]. The *Candida albicans* MDM8 strain was donated by Instituto de Ciências Biomédicas from Universidade de São Paulo. The *E*. *coli* D31 *and Enterobacter cloacae* β12 strains were kindly donated by Dr. Hans G. Boman group from University of Stockholm. The *M*. *luteus* BR2 strain was donated by Faculdade de Ciências Farmacêuticas from Universidade de São Paulo. The yeast strains from clinical isolates deposited in the Oswald Cruz Institute collection were kindly provided by Prof. Mirian Hayashi (Pharmacology Department–Federal University of São Paulo): *Candida albicans* IOC4558, *Candida tropicalis* IOC4560 and *Candida guilliermondii* IOC4557. *Staphylococcus aureus* ATCC29213, *Staphylococcus epidermidis* ATCC12228, *Pseudomonas aeruginosa* ATCC27853, *Serratia marcescens* ATCC4112 and *Cladosporium herbarum* ATCC 26362 were acquired from the American Type Culture Collection (http://www.atcc.org). *Aspergillus niger* was isolated from bread. *Paecilomyces farinosus* IBCB251 is an entomopathogenic filamentous fungus obtained from the “Oldemar Cardim Abreu” collection of the Instituto Biológico (IB-CB).

### 2.4 Bioassays

#### 2.4.1 Microbial growth inhibition assays

HPLC fractions were concentrated under a vacuum centrifuge and resuspended in 50–100 μL of deionized water. Antimicrobial activity from the HPLC fractions was evaluated against *M*. *luteus* A270 in a liquid growth inhibition assay previously described by Bulet et al. [27] in 96-well plates. Briefly, 10 μL of the sample was added to 90 μL of bacteria in poor peptone broth (PB: peptone 1% and NaCl 86 mM, pH 7.4) in mid-log phase (OD_600nm_ ≈ 0.6). To evaluate microbial growth, the OD_595nm_ was measured on a Victor 3 (Applied Biosystems, USA) microplate reader after 18 hours under shaking at 30°C.

Minimal inhibitory concentration (MIC) of the synthetic peptide was evaluated under poor broth conditions using PB or Poor Dextrose Broth (PDB: 1.2% potato dextrose, pH 5) for bacteria or fungi, respectively. Briefly, the bacteria (~10^5^ CFU/ml) and the yeasts or filamentous fungi (~10^3^ CFU/ml) were inoculated in 90 μl of broth with 10 μl of water (negative control) or a stock solution of the synthetic peptide in serial two-fold dilutions starting from 120 μM as the highest final concentration. To evaluate microbial growth, bacteria or fungi plates were incubated at 30°C for 18 or 48 hours, respectively. MICs values are expressed as a range [**A**]–[**B**], where **B** was the highest peptide concentration at which microbial growth was similar to negative controls and **A** was the lowest concentration that visually inhibited the growth. MICs values of streptomycin, a conventional antimycobacterial drug, were also obtained in a serial two-fold dilutions method starting from 2000 μg/ml concentration or used at 2000 μg/ml concentration as a positive control for inhibition of microbial growth.

#### 2.4.2 MTT cytotoxicity assay

Vero cells were cultured on L-15 medium, supplemented with 10% Fetal Bovine Serum (FBS) at 37°C in a humidified atmosphere containing 5% CO_2_. The cells were cultured in T-25 flask had a 25 cm^2^ growth area.

Vero cells were seeded in 96-well F-bottom plates at a density of 6 x 10³ cells/well. After confluent growth by 70–80% of the cell carpet, the culture medium was removed and the treatments began with the sample. The control wells received 100 μl of culture medium with 0% or 10% FBS. For the treated wells, 20 μl of longipin 2 mM solution was diluted in 180 μl of L-15 medium and a serial two-fold dilution was conducted to give a final volume of 100 μl per well at a highest longipin final concentration of 200 μM. After 24 hours of treatment, the supernatant was carefully removed and added 20 μl of MTT (5 mg/ml on L-15 medium) to each well and incubated 3 hours at 37°C. The formazan crystals were solubilized by the addition of 100 μl per well of DMSO and the absorbance was measured at a 540 nm wavelength. The average reading of control was regarded as 100% cell viability being compared with the average for each treatment.

### 2.5 Mass spectrometry

Matrix-assisted laser desorption ionization mass spectrometry (MALDI-ToF-MS) was performed in an Ettan MALDI-ToF/Pro spectrometer (Amersham Biosciences) operating in reflectron mode. A total of 0.35 μL of the sample was mixed with the same volume of the matrix (saturated solution of α-cyan-hydroxycinnamic acid in ACN/H_2_O in a 1:1 ratio) and allowed to dry prior to the analysis.

Electrospray ionization mass spectrometry (ESI-MS) was performed on a Surveyor MSQ Plus (Thermo Fisher Scientific) spectrometer coupled to a Surveyor HPLC. The system was previously equilibrated with ACN/H_2_O (1:1) and 0.1% formic acid at a 0.5 ml/min flow rate prior to sample injection (10 μL). The electrospray temperature was set to 350°C and the cone voltage to 3 kV. Scans were recorded at a 2 s^-1^ rate. The final spectrum was obtained using the Xcalibur 2.0.7 software (Thermo Fisher Scientific), and *m/z* value deconvolution was performed with the MagTran 1.02 software [28].

LC-Q-ToF-MS/MS was performed with a Q-ToF Ultima™ API (Micromass) spectrometer coupled to a nanoAcquity UPLC (Waters) system. Samples (4.5 μL) loaded into the HSS T3 (1.8 μm, 150 μm × 100 mm) column (Waters) were eluted in a 5–80% gradient of ACN/0.1% formic acid for 30 min with a 1.1 μL/min flow rate. The source temperature was set to 70°C and the cone voltage to 50 eV. During MS scan acquisition, the collision energy (CE) was set to 10; for ion fragmentation, the CE were based on a previous work from Mouls et al. [29].

### 2.6 Peptide Sequencing

#### 2.6.1 Reduction and alkylation

An 8 μL aliquot of the native peptide from the HPLC fraction was dried under a vacuum centrifuge and resuspended in 20 μL of 400 mM NH_4_HCO_3_ and 8 M urea. A total of 5 μL of 45 mM DTT was added prior to incubation at 50°C for 15 min. After chilling, 5 μL of 100 mM iodoacetamide was added, and the solution was kept for 15 min at room temperature protected from light. The product was subjected to desalting using Zip Tip® C18 (Applied Biosystems, USA) columns prior to MALDI-ToF-MS analysis.

#### 2.6.2 Acetylation

Chemical modification of the Lys side chain was performed with an acetylation protocol [30] to differentiate Lys residues (128.09 Da) from Gln residues (128.03 Da) in the CID spectra. Briefly, 5 μL of the native peptide from the HPLC fraction was dried under a vacuum centrifuge and resuspended in 20 μL of 50 mM NH_4_HCO_3_ and 50 μL acetic anhydride/methanol (1:3). This solution was incubated for one hour at room temperature prior to enzymatic digestion.

#### 2.6.3 Enzymatic digestion

An 8 μL aliquot of the peptide from analytical HPLC or the sample subjected to the acetylation protocol (section 2.6.2) was lyophilized and resuspended in 100 μL of 100 mM NH_4_HCO_3_ (pH 7.8). A total of 50 ng of the endoprotease Glu-C V8 (Sigma-Aldrich) was added, and the solution was kept for 8 h at 37°C prior to the addition of 100 μL of 0.01% TFA to stop the reaction. The samples were lyophilized, resuspended in 40 μL of 0.05% TFA and subjected to desalting using Zip Tip® C18 columns prior to LC-Q-ToF-MS/MS analysis.

#### 2.6.4 ‘De novo’ peptide sequencing

Collision-induced dissociation (CID) spectra from peptides obtained by LC-Q-ToF-MS/MS were processed using the MaxEnt3 tool (MassLynx 4.1 software, Waters). The *-y* and *-b* ions series [31] were manually interpreted and marked in the spectra.

#### 2.6.5 N-terminal sequencing

To differentiate the Ile/Leu residues, the peptide (5 μL from the HPLC peptide fraction) was subjected to Edman degradation using a PPSq 21 Automated Protein Sequencer (Shimadzu Co, Japan).

### 2.7 Peptide synthesis

The peptide was manually synthesized by standard Fmoc solid phase peptide synthesis (SPPS) technology [32] using 50 mg of Fmoc-Phe-TGA resin (Novabiochem Inc.) with a 0.24 mmol/g substitution rate.

Fmoc deprotections were performed with a dimethylformamide (DMF)/morfolin/1,8-diazabicycloundec-7-ene (DBU) (49:49:2) solution (2 × 7 min). Fmoc-amino acids (5 equivalents with respect to the peptide-resin) were coupled to the growing sequence in 460 μL of DMF/N-methylformamide (NMF) (67:13) with TBTU (40 mg) solution for 1.5 hours. The couplings were evaluated by the Kaiser test [33] and/or MALDI-ToF-MS analysis.

Cleavage from the resin and the removal of side chain-protecting groups were simultaneously performed with 1 ml of TFA/phenol/H_2_O/ethanedithiol (EDT) (82.5:5:5:5:2.5) for 12 hours; then, the sample was dried under an N_2_ steam. Crude peptides were precipitated in chilled diethyl ether, resuspended in DMF and purified by preparative RP-HPLC with a Shim-pack PREP-ODS column (5 μm, 20 × 250 mm). The resultant fractions were analyzed by MALDI-ToF-MS to evaluate their homogeneity.

Final peptide homogeneity/purity was determined by RP-HPLC using a Shim-pack VP-ODS column (5 μm, 4.6 × 250 mm) and by ESI-MS. The CID spectra of synthetic and native peptides were compared using the mMass 3.9 software [34].

### 2.8 Large unilamellar vesicle preparation

Large unilamellar vesicles (LUVs) were prepared with 1-palmitoyl-2-oleoyl-sn-glycero-3-phosphocholine (POPC), 1-palmitoyl-2-oleoyl-sn-glycero-3-phosphoglycerol (POPG) or POPG:POPC (1:1 molar ratio) phospholipids purchased from Avanti® Polar Lipids (Alabaster, USA). Lipids were dissolved in chloroform/methanol (2:1, v/v), dried under N_2_ steam and kept overnight under vacuum. Then, the samples were resuspended in H_2_O with the desired NaCl concentration. LUVs were prepared by five freeze-thaw cycles (liquid nitrogen and 40°C) and extruded through 100 nm pore polycarbonate filters [35] with an extrusion device from Avestin (Ottawa, Canada).

To prepare LUVs for thioflavin-T binding assay, lipids were dissolved in chloroform, dried under N_2_ steam and kept for 2 h under vacuum. Lipids were then resuspended in 30 mM HEPES buffer pH 7.4 and extruded at least 11 times through 100 nm pore polycarbonate filters with an extrusion device from Avanti® Polar Lipids.

### 2.9 Filtration binding assay

Samples containing the peptide and peptide-LUV in a 1:50 peptide/lipid (P/L) molar ratio in 10 mM NaCl were incubated for 30 min at room temperature and loaded into the upper chamber of 100 kDa Amicon Ultra 0.5 ml centrifugal devices (Millipore, EUA). Centrifugation was performed at 4000 × g for 10 min at room temperature, and the peptide was detected by Tyr fluorescence (λ_EX_ = 275 nm / λ_EM_ = 302 nm) in the filtered solution in a SLM Aminco 4800C (Jovin Yvon Horiba, Japan) fluorometer.

### 2.10 Titration assay

Titrations of 20 μM peptide solutions in four different NaCl concentrations (1, 10, 50 and 200 mM) with LUVs were conducted under increased P/L molar ratios (1:0, 1:1, 1:5, 1:10, 1:20, 1:50 and 1:100) by monitoring the fluorescence of the peptide (λ_EX_ = 275 nm / λ_EM_ = 302 nm). The results were expressed by the *F*/*F0* ratio, where *F* and *F0* are arbitrary values of fluorescence in the presence or absence of vesicles, respectively. Exponential adjustment of the data was used to evaluate the apparent dissociation constant (*Kd*) of the peptide-vesicle binding equilibrium.

### 2.11 Dye leakage assay

Carboxyfluorescein (CF)-loaded vesicles were extruded in 80 mM CF and then subjected to size exclusion filtration to eliminate free carboxyfluorescein molecules in a manually packed Sephadex G-75 resin column equilibrated with 80 mM NaCl. The bee venom antimicrobial peptide melittin (Sigma-Aldrich) was used as a positive control of a membrane active peptide.

The fluorescence CF-loaded vesicles samples (160 μl) were monitored (λ_EX_ = 474 nm / λ_EM_ = 518 nm) in a SLM Aminco 4800C (Jovin Yvon Horiba, Japan) fluorometer during 3000 s. After 200 s, 40 μl of longipin or melittin solution were added to achieve a 20 μM peptide concentration in the sample. The same volume of buffer was added for a negative control assay. Triton X-100 was added at a 0.1% (v/v) final concentration after 2500 s to achieve the maximum fluorescence intensity.

### 2.12 FT-IR spectroscopy

Fourier-transformed infrared spectra (FT-IR) were acquired in a Nicolet Nexus (Thermo, USA) spectrometer equipped with a holder for liquid samples in CaF_2_ windows and 75 μm spacers. The spectrometer was purged with dried N_2_ for 1 h prior to spectra acquisition, and the sample holder temperature was kept at 25°C.

Samples were prepared with 0.6 mg of peptide resuspended in 70 μl of 10 mM NaCl in deuterium oxide (D_2_O). For FT-IR spectroscopy, we used multilamellar vesicles. Lipids were dried from chloroform/methanol (2:1) and resuspended with a solution containing the peptide and 10 mM NaCl in D_2_O. A total of 100 scans were collected and averaged from the samples and backgrounds. The resolution was 2 cm^-1^. The spectra of pure buffer were subtracted from the samples. The resulting spectra were Fourier self-deconvolved using a bandwidth of 18 cm^-1^ and a narrowing factor *k* = 2. The position of the component bands was obtained by the second derivative of the deconvoluted spectra. The proportion of the component bands was obtained by fitting to the original (not deconvoluted) spectra according to the procedures described by Arrond and Goòi [36] and Nolan et al. [37]. Assignment of secondary structures was performed according to Byler and Susi [38] and Chiti et al. [39].

### 2.13 Thioflavin-T binding assay

To monitor thioflavin-T (ThT) fluorescence intensity change, a emission spectra (λ_EX_ = 440 nm) of a 10 μM ThT solution in 30 mM HEPES buffer (pH 7) was initially acquired. Then, a stock solution of peptide (600 μM) and/or POPG:POPC (1:1 molar ratio) or POPC LUVs (3 mM lipid) was added to a final concentration of 4 and 80 μM, respectively, to obtain a final 1:20 peptide:lipid molar ratio. Emission spectra were then acquired after incubation for 1, 2, 3, 4, 5, 7, 10, 15, 20 and 30 min at room temperature. ThT fluorescence was normalized at the maximum emission intensity (486 nm) over initial values.

## 3 Results

### 3.1 Peptide purification

**Fig 1:**
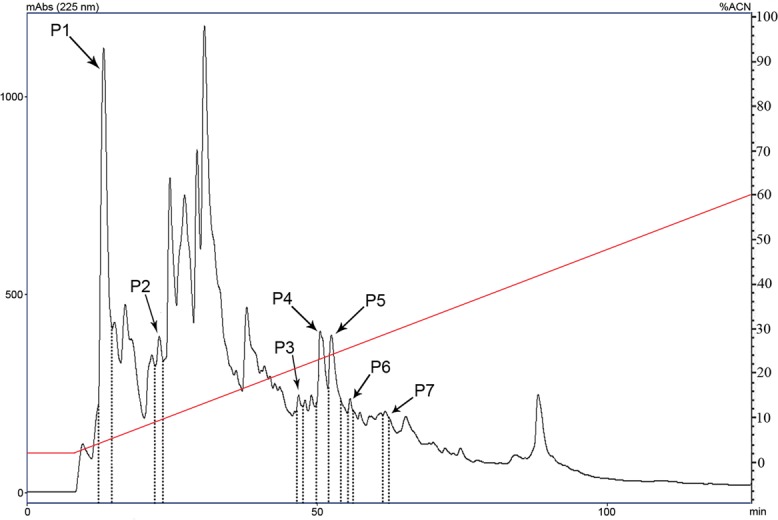
First HPLC step purification of harvestmen plasma. Antimicrobial fractions obtained from first step purification by RP-HPLC of the fraction from harvestmen plasma eluted with 40% ACN from a Sep-Pak cartridge. The chromatography was performed in a semi-preparative Vydac C18 (5 μm, 250 × 10 mm) column with a linear gradient from 20% to 80% of ACN/0.05% TFA in H_2_O/0.05% TFA for 120 min at a 1.5 ml/min flow rate. Arrows indicate the fractions that showed anti-*M*. *luteus* activity. Abs was monitored at 225 nm.

The material from the plasma extract eluted with 40% ACN in the Sep-Pak cartridge was subjected to RP-HPLC on a semi-preparative column (Fig 1). All fractions were dried, resuspended in water and used in the antimicrobial growth inhibition assay (section 2.4) against the Gram-positive bacteria *M*. *luteus* A270. Seven of these fractions showed anti-*M*. *luteus* activity (**P1-P7**; Fig 1).

All active fractions obtained from plasma were subjected to a second purification step by RP-HPLC under optimized ACN gradients. The fractions with antimicrobial activity were revaluated against *M*. *luteus*. Each chromatography resulted in at least one active fraction, and most of them presented MALDI-ToF/MS spectra with *m/z* values between 1,000 and 10,000 Da (data not shown).

**Fig 2:**
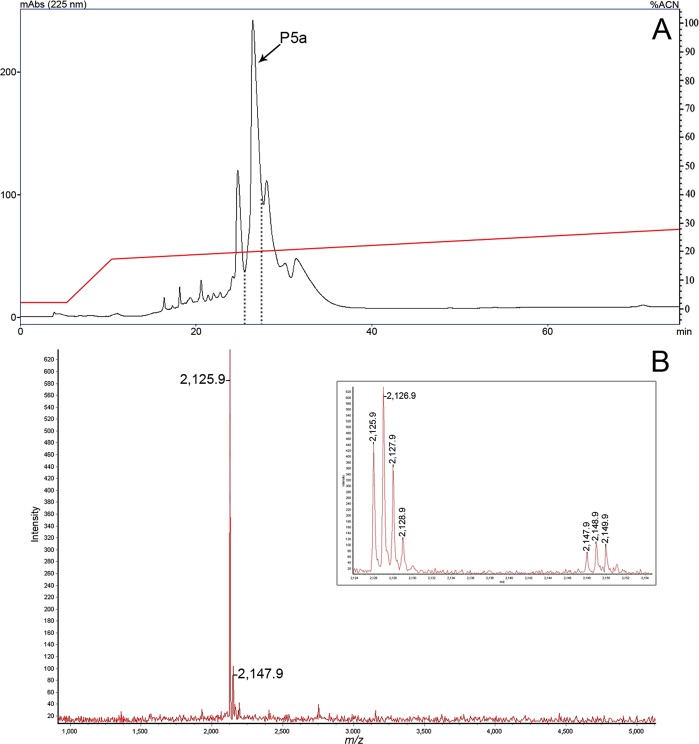
Second step purification and MALDI-ToF analysis of the P5 fraction. (A) RP-HPLC was performed with an analytical Vydac C18 (5 μm, 250 × 4.6 mm) column with an optimized linear gradient from 18% to 28% ACN/TFA 0.05% in H_2_O/TFA 0.05% for 60 min at a 1.0 ml/min flow rate. Fraction **P5a** showed anti-*M*. *luteus* activity. (B) MALDI-ToF spectrum of fraction **P5a** showing its homogeneity. The single detected peptide was charged with a proton ([M+H]^+^) at *m/z* 2124.9 and with a sodium adduct ([M+Na]^+^) at *m/z* 2147.9. The inset presents the details of the isotopic patterns of these *m/z* values.

Among these fractions, **P5a** (Fig 2A), obtained after a second purification step of **P5**, was the only fraction that presented a single peptide (2.1 kDa) by MS analysis. Therefore, we assumed that this peptide was responsible for the anti-*M*. *luteus* activity and aimed to elucidate its primary structure. The MALDI-ToF spectrum showed two *m/z* values with mono-charged isotopic patterns (Fig 2B inserted) that differed by 22 Da, which indicated the presence of a 2124.9 Da protonated peptide ([M+H]^+^ = 2125.9 Da) and its sodium adduct charged form ([M+Na]^+^ = 2147.9 Da).

### 3.2 “De novo” sequencing

After reduction and alkylation **P5a** fraction peptide, no mass increment was observed in the 2.1 kDa peptide (data not shown), suggesting the absence of Cys residues.

**Fig 3:**
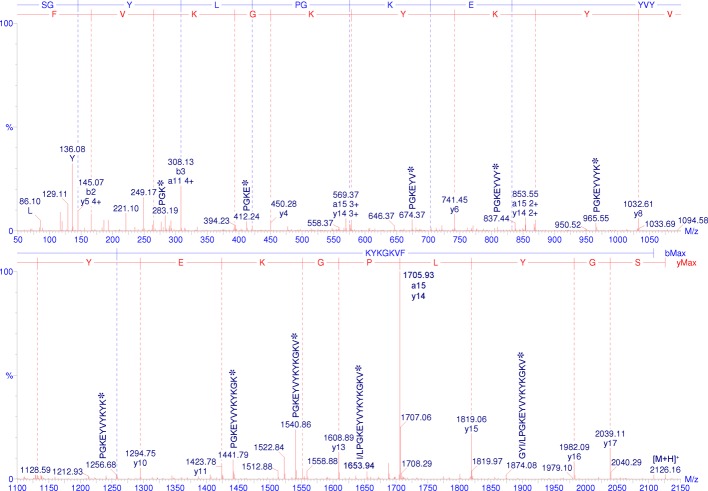
“De novo” sequencing by Q-ToF-MS/MS of the peptide from the P5a fraction. The CID spectrum from the 4+ ion ([M+4H]^4+^, *m/z* 532.27) was acquired under a 20 V potential in the collision cell. The original spectrum was processed with the MaxEnt3 tool (MassLynx 4.1) to convert the multi-charged ions into mono-charged ions. The -*y* and -*b* fragments are marked on top of the figure in red and blue, respectively. Several internal fragments and their sequences (√) are also marked on the spectrum. Lys and Gln differentiation was not achieved because the equipment mass accuracy (±0.03 Da) was in the same range of the mass difference (≈0.03 Da) of these residues. Differentiation between Leu and Ile is not possible through this methodology because they are isobaric residues (113.08 Da).

The primary structure of this peptide was deduced from the processed CID spectrum of the 4+ ion ([M+4H]^4+^, *m/z* 532.27) (Fig 3). All of the *-y* (blue) and seven -*b* (red) fragments were marked on the top of the spectrum, revealing a peptide composed of 18 amino acid residues (SGYI/LPGK/QEYVYK/QYK/QGK/QVF). The differentiation of the isobaric residues Ile and Leu and between residues with similar masses (Lys and Gln) could not be achieved by this methodology. The presence of Ser, Pro, Val, Ile/Leu, Phe and Tyr immonium ions and Lys/Gln related ions (data not shown) [40] was in agreement with the deduced sequence. In the CID spectra, we could also observe several internal ion fragments (√), most of which had an N-terminal Pro residue because their N-terminal peptide bonds has a high tendency to break upon CID mass spectrometry analysis [41].

Differentiation between Lys and Gln residues was achieved after chemical modification of the peptide by an acetylation protocol (42 Da mass increment in the Lys side chain amino group and its free N-terminus), followed by enzymatic cleavage with the endoprotease Glu-C. Due to the presence of one Glu residue in the primary sequence, two digestion fragments with 933.18 Da and 1419.6 Da were observed by ESI-MS (Figure A in S1 File). MS/MS “de novo” sequencing of the 933 Da fragment (Figure B in S1 File) revealed that this was the N-terminal fragment modified by two acetyl groups (849+2×42 = 933 Da): the N-terminal Ser and the Lys side chain amino group (^AC^SGYI/LPGK^AC^E). The 1419 Da fragment contained the C-terminus of the peptide with three acetyl modifications (1293+3×42 = 1419 Da) in Lys residues (YVYK^AC^YK^AC^GK^AC^VF) (Figure B in S1 File). An ammonia neutral loss from the acetyl-lysine immonium ion (K^AC^-NH_3_) was detected at *m/z* 126 [42] in the low mass regions of both spectra (Figure B in S1 File). These results showed the presence of four Lys and no Gln residues in the primary sequence of this peptide.

The partial primary sequence determined by Edman degradation (SGYLPGK) was in agreement with the sequence deduced from the MS/MS spectra. Moreover, it showed a Leu residue at the fourth position.

**Fig 4:**
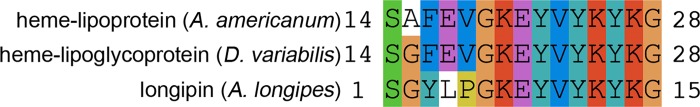
Sequence similarities between longipin and heme-lipoproteins from the ticks *Amblyomma americanum* (GenBank: ABK40086.2) and *Dermacentor variabilis* (GenBank: ABD83654.1).

This peptide, composed of 18 residues (SGYLPGKEYVYKYKGKVF), was named longipin. It is positively charged (+3) at neutral pH and presented high pI value (9.52). Longipin primary sequence showed no similarity with other AMPs. However, the first 15 residues showed high similarity with heme-lipoproteins from *Dermacentor variabilis* and *Amblyomma americanum* ticks (Fig 4).

### 3.3 Synthetic longipin

Homogeneity of synthetic longipin was determined by relative peak area obtained after an analytical chromatography. The major peak was collected and analyzed by ESI-MS. Deconvolution of the *m/z* values from the spectrum showed the expected average mass of longipin (2127.5 Da). Ions from the K^+^ adduct (2166.6 Da) were also detected. The synthetic peptide purity was around 90% (Figure C in S1 File).

The similarity between the synthetic (green) and native (blue) longipin CID spectra (Figure D in S1 File), with the exception of the intensity of the ions at *m/z* 1593.8 and the immonium ions in the low *m/z* region, confirmed the deduced sequence from MS/MS spectrum.

### 3.4 Longipin activity spectrum

Synthetic longipin presented antimicrobial activity against Gram-positive (*M*. *luteus*) and Gram-negative (*Pseudomonas aeruginosa* and *Serratia marcescens*) bacteria and *Candida sp*. yeasts (*C*. *albicans*, *C*. *tropicalis* and *C*. *guilliermondii*) (Table 1). The peptide did not show activity against the filamentous fungi evaluated. All minimal inhibitory concentration (MIC) intervals were in the μM range and increased activity was obtained against *C*. *tropicalis* and *C*. *guilliermondii* yeasts (MIC: 3.8–7.5 μM). Streptomycin at 2000 μg/ml, used as a positive control, completely inhibited the growth of all microorganisms. MIC ranges for this antimycobacterial drug were also determined (Table A in S1 File).

**Table 1:** Spectrum of activity and MIC values of synthetic longipin.

Microorganisms	MIC (μM)
**- Gram-positive bacteria**	
*Staphylococcus aureus* ATCC29213	ND
*Staphylococcus epidermidis* ATCC12228	ND
*Micrococcus luteus* BR2	ND
*Micrococcus luteus* A270	60–120 (126–252 μg/ml)
**- Gram-negative bacteria**	
*Pseudomonas aeruginosa* ATCC27853	60–120 (126–252 μg/ml)
*Escherichia coli* D31	ND
*Escherichia coli* SBS363	ND
*Serratia marcescens* ATCC4112	60–120 (126–252 μg/ml)
*Enterobacter cloacae* β12	ND
**- yeasts**	
*Candida albicans* MDM8	15–30 (31.5–63 μg/ml)
*Candida albicans* IOC4558	7.5–15 (15.8–31.5 μg/ml)
*Candida tropicalis* IOC4560	3.8–7.5 (7.9–15.8 μg/ml)
*Candida guilliermondii* IOC4557	3.8–7.5 (7.9–15.8 μg/ml)
**- filamentous fungi**	
*Aspergillus niger*	ND
*Cladosporium herbarum* ATCC26362	ND
*Paecilomyces farinosus* IBCB251	ND

**ND-** not detected at the highest concentration tested (120 μM).

Mammalian cells viability was evaluated by MTT assay. Longipin did not interfered with VERO cells viability after 24 h treatment at different peptide concentrations (200–0.1 μM range) (Figure E in S1 File).

### 3.5 Interaction with phospholipid vesicles

**Fig 5:**
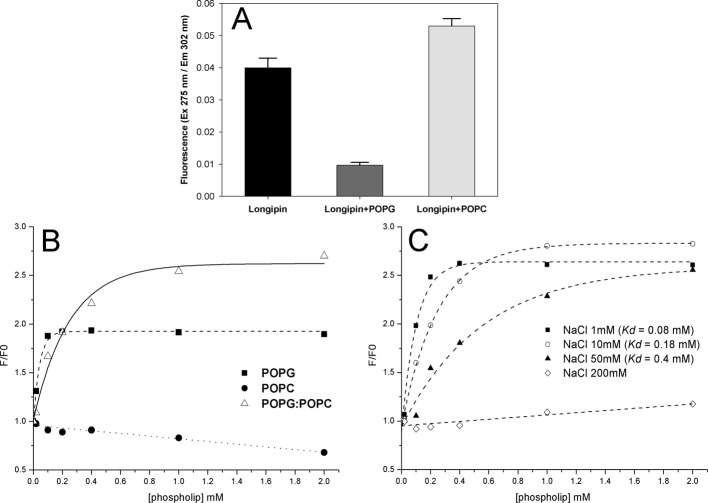
Binding of longipin to lipid membranes. (**A**) Filtration binding assay of longipin binding to LUVs composed of POPC or POPG. The peptide was detected in the filtrate by monitoring the fluorescence of Tyr residues (λ_EX_ = 275 nm / λ_EM_ = 302 nm). (**B**) Titration of 20 μM longipin with LUVs composed of POPG, POPC and POPG:POPC (1:1 molar ratio). (**C**) Titrations at different NaCl concentrations in the 20 μM longipin solution with POPG:POPC LUVs. Dissociation constants (*Kd*) were determined for each condition.

The interaction of longipin with LUVs was initially evaluated with the filtration binding assay. This assay showed preferential binding to anionic POPG vesicles (Fig 5A), indicating that electrostatic forces between the peptide and negatively charged vesicles might drive this interaction. In agreement with this result, titration experiments showed that the intrinsic fluorescence of longipin increased only upon the addition of negatively charged LUVs (POPG or POPG:POPC; Fig 5B). The electrostatic binding force dependence was also verified through titration with POPG:POPC vesicles in solutions with different NaCl concentrations (Fig 5C). Dissociation constants (*Kd*) increased with the ionic strength, showing that the peptide-LUV affinity decreased with the shielding of charges by Na^+^ and Cl^-^ ions in solution.

FT-IR spectroscopy was used to evaluate structural changes in longipin upon binding to multilamelar vesicles (MLVs). The amide I’ region (1600–1700 cm^-1^) reports the stretching frequencies of carbonyl groups, which are mainly associated with backbone conformations [38].

**Fig 6:**
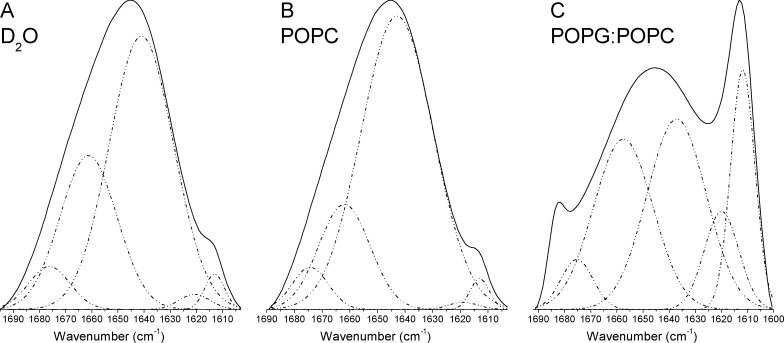
Peak fitting of amide I’ region FT-IR spectra of longipin. Amide I’ band of original (not deconvoluted) FT-IR spectra (continuous lines) and their fitted bands (dashed lines) of longipin in solution (D2O) and in the presence of MLVs composed of POPC or POPG:POPC (1:1 molar ratio).

Longipin chain was mainly disordered in solution or in the presence of zwitterionic POPC vesicles (Fig 6; D_2_O and POPC) according to the observed maximum at 1643 cm^-1^ in the amide I’ region of the FT-IR spectra. However, in the presence of anionic mixed POPG:POPC (1:1 molar ratio) vesicles, the spectrum showed a maximum centered at 1613 cm^-1^ and an increased contribution of 1683 cm^-1^ band (Fig 6; POPG:POPC). This spectrum is characteristic of intermolecular aggregates in amyloid fibrils, which are structured in a β-sheet-like conformation called cross-β [39, 43].

Peaks assigned in the deconvoluted spectra (Figure F in S1 File) were used to estimate the secondary structure contents under each condition (in solution or in the presence of MLVs) after fitting the original FT-IR spectra [36]. The fitted spectra (Fig 6) confirmed that longipin was mainly disordered in solution (73%) and in the presence of POPC MLVs (79%), but considerable β-turn contents and smaller contributions of β-sheet and amyloid aggregates were also present under these conditions (Table 2), indicating the presence of structured regions of the peptide.

**Table 2:** Secondary structure contents in the amide I’ region of longipin in solution (D_2_O) and in POPC or POPG:POPC multilamelar vesicles. Numbers in parenthesis indicate the contribution of fitted Gaussians to the original spectra.

	Assigned frequencies in amide I’ region (cm^-1^)				
	α-helix ^a^	β-turn ^a^	β-sheet ^a^	Amyloid aggregates ^b^	disordered ^a^
**D**_**2**_**O**	---	1661 (30%)	1621,1676 (8%)	1613 (2%)	1641 (60%)
**POPC**	---	1662 (19%)	1619, 1674 (6%)	1613 (2%)	1643 (73%)
**POPG:POPC**	---	1658 (30%)	1620, 1637, 1675 (50%)	1612, 1683 (20%)	---

**a-** according to Byler and Susi (1986)

**b-** according to Chiti et al. (1999)

Longipin became structured upon binding to anionic POPG:POPC MLVs, where amide I’ bands characteristic of conformational disorder (1642–1646 cm^-1^) were absent. Higher contents of β-turn (30%), β-sheet (50%) and amyloid aggregates (20%) compared to D_2_O (Table 2) showed that longipin binding to anionic vesicles was mediated by a disorder-to-order transition of the peptide chain.

ThT is a potent fluorescent marker of amyloid fibrils that preferentially binds to side chain channels formed along the principal axis of fibrils [44]. Upon fibrils binding. ThT fluorescence is enhanced and thus it became an important dye for the detection of amyloid and amyloid-like structures.

**Fig 7:**
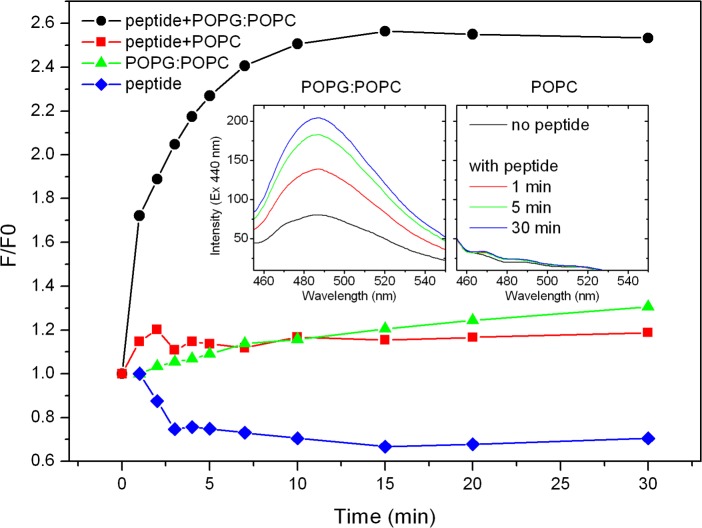
Longipin amyloid-like structures formation evaluated by thioflavin-T fluorescence assay. Fluorescence intensity of ThT 10 μM solution in HEPES buffer (30 mM; pH 7.4) was monitored (λ_EX_ = 440 nm / λ_EM_ = 486 nm) in the presence of longipin 4 μM (blue-dotted line), longipin and POPG:POPC (black-dotted line) or POPC (red-dotted line) vesicles in a 1:20 peptide:lipid molar ratio. The inlet shows representative emission spectra of longipin and POPG:POPC or POPC vesicles incubated for 1, 5 and 30 min.

Fluorescence intensity of ThT at 486 nm of longipin in solution (blue-dotted line; Fig 7) or in the presence of zwitterionic POPC vesicles (red-dotted line; Fig 7) did not significantly increase over time. However, an evident ThT fluorescence enhancement was observed when longipin was bounded to negatively charged POPG:POPC (1:1 molar ratio) vesicles, reaching equilibrium values after ~15 min (black-dotted line; Fig 7) and indicating that longipin binding to negatively charged vesicles in accompanied by peptide chains folding into amyloid-like conformations. A modest raise in ThT fluorescence intensity was also observed in a solution of POPG:POPC vesicles (green-dotted line; Fig 7), indicating that part of the ThT fluorescence gain after longipin binding to POPG:POPC vesicles might occur due to partitioning of the dye to the phospholipid bilayer. Thus, ThT assay was in agreement with FTIR spectroscopy that showed an increased content of amyloid-like structures in longipin bounded to negatively charged vesicles (POPG:POPC) when compared to the peptide free in solution or in the presence of zwitterionic vesicles (POPC).

**Fig 8:**
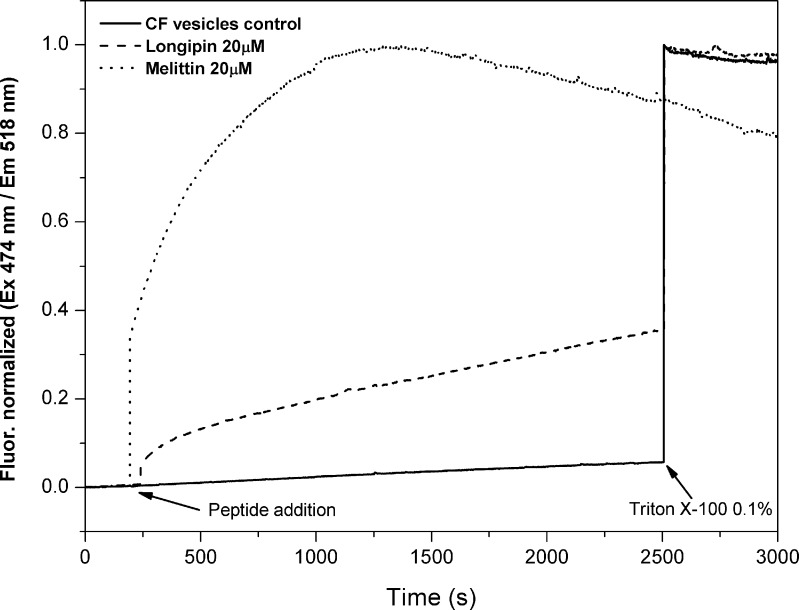
Dye leakage induced by longipin. Dye leakage assay from CF-loaded POPG:POPC vesicles in 80 mM NaCl. Longipin (traced line), melittin (dotted line) or buffer (continuous line) were added at ~ 200 s to a final concentration of 20 μM. CF fluorescence was monitored (λ_EX_ = 474 nm / λ_EM_ = 518 nm), and Triton X-100 was added to a final 0.1% concentration to achieve the maximum fluorescence intensity.

To investigate the influence of longipin binding in the bilayer permeability, the peptide was added to a solution of POPG:POPC CF-loaded vesicles. This assay explores the self-quenching effect between CF molecules at high concentrations (80 mM) trapped inside vesicles. When membrane permeability increases, trapped CF molecules can diffuse into the solution, thereby decreasing the self-quenching effect and consequently increasing its fluorescence. This phenomenon was observed after the addition of longipin to a solution of CF-loaded vesicles (Fig 8), suggesting that the phospholipid membrane permeability was increased. In this experiment, melittin was used as a positive control of a membrane active AMP that forms pores in artificial phospholipid membranes [45]. Longipin also showed a dose-dependent effect on dye leakage of negatively charged vesicles when increasing concentrations of peptide (1, 5, 10 and 20 μM) were used to evaluate CF release from POPG:POPC vesicle (Figure G in S1 File).

## 4 Discussion

In this work, we presented the purification, characterization, spectrum of biological activity and vesicle interaction studies of a new antimicrobial peptide isolated from the hemolymph of the harvestman *Acutisoma longipes*.

Several fractions with antimicrobial activity were obtained from the hemolymph of unchallenged harvestmen that had been kept under aseptic conditions for 10 days prior to hemolymph extraction. This fact indicates the constitutive expression of AMPs in the immune system of Opiliones, which is similar to several Chelicerata orders (e.g., Aranae [6–8, 15, 46], Scorpiones [9] and Xiphosura (horseshoe crabs) [3, 5]). Furthermore, it is also in agreement with the assumption that the constitutive expression of AMPs is a characteristic present in all lower invertebrate groups compared to the immune system complexity of higher insects, which trigger the expression of AMPs after microbial infection [4].

After two reverse-phase chromatography of the plasma acid extract, we obtained a homogeneous fraction that presented a single peptide with 2.1 kDa. Its aminoacid sequence was elucidated by MS/MS spectrum analysis and N-terminal sequencing by Edman degradation methodology. Due to the spectrometer *m/z* resolution window, differentiation between Lys and Gln residues was only achieved after peptide acetylation and enzymatic cleavage (Figures A and B in S1 File).

Longipin presented physicochemical proprieties of a cationic antimicrobial peptide, including a positive net charge (+3) and high pI value (9.52). The synthetic and native longipin CID spectra were in agreement (Figure D in S1 File), supporting the deduced primary structure.

Sequence similarities were observed between longipin and heme-lipoproteins (HeLp) from two tick species (Fig 4). HeLp is the major constituent of *Rhipicephalus* (*Boophilus*) *microplus* hemolymph and has an important role in heme (obtained from blood feeding) binding and transport to other tissues [47] because the heme biosynthetic pathway is absent in these arachnids [48]. This sequence identity suggests that longipin might be generated from a precursor protein similarly to observed in the tarantula *Acanthoscurria rondoniae* [18] and *Penaeus* sp. shrimp [16], where cleavage of the hemocyanin C-terminal region originate peptides with antimicrobial activity. However, further investigations should be conducted to evaluate this hypothesis.

Selectivity of AMPs that are only active against microorganisms is related to differences between the net charge of microbial (negatively charged) and mammalian (zwitterionic) cell membrane surfaces [49]. Therefore, preferential binding of longipin to negatively charged POPG:POPC vesicles is in agreement with the absence of activity against mammalian VERO cells line verified by MTT viability assay and points to its selectivity against microbial cells. This selectivity is important for the development of AMP-based antibiotics [50–52].

FTIR spectroscopy and thioflavin-T fluorescence assay indicated that longipin adopted amyloid-like fibrils conformation, similarly to other amyloid antimicrobial peptides [23, 53, 54], upon binding to anionic vesicles. This result reinforces the link between the mode of action of amyloid antimicrobial peptides and amyloid-related pathogenesis [22], where amyloid aggregates are thought to interfere with the plasma membrane permeability. Wu et al showed by molecular dynamics simulations that thioflavin-T preferentially binds to a Tyr-Leu groove parallel the principal axis of a peptide self-assembly mimic of amyloid fibers [55]. Interestingly, the same Tyr-Leu motif is present in the primary sequence of longipin (SG**YL**PG…), indicating a probable binding site for ThT.

The presence of the band centered at 1614 cm^-1^ together with a high contribution of disordered peak (1643 cm^-1^) in the FT-IR spectrum of longipin in vesicles-free medium or in POPC might suggest the existence of a low population of peptide molecules in amyloid aggregate conformation. This hypothesis is in agreement with the considerable amount of β-turn and β-sheet–that could be part of amyloid structures–also observed under these conditions. Therefore, addition of anionic vesicles would shift the equilibrium between disordered and amyloid structures of longipin in solution, increasing the population of amyloid aggregates. In this sense, it would be interesting to verify the influence of anionic vesicles addition in amyloid-like aggregates folding kinetics.

We also showed that the longipin chain in solution or in the presence of POPC was mainly disordered but also presented a high β-turn content, which is in agreement with the presence of Pro-Gly motif in the primary structure, that would favor formation of a type II β-turn. Moreover, prediction by NetTurnP method [56] pointed to the presence of a β-turn in the segment Leu_4_-Pro_5_-Gly_6_-Lys_7_, which corresponds to 22% of the peptide chain. In quantitative agreement with this result, FT-IR spectrum of longipin in solution or POPC respectively presented 30% and 19% of β-turn content (Table 2).

Dye leakage from CF-loaded vesicles suggests that the permeability of the vesicles were increased after longipin binding. This result suggests that longipin is a membrane active AMP that targets the plasmatic membrane of microorganisms.

Lüders et al. [57] showed that the proline isomerization state (*cis* or *trans*) at the N-terminal peptide bond influenced the activity of a proline-rich antimicrobial peptide. The authors could only observe antimicrobial activity in the synthetic peptide after incubation with the peptidylproline *cis-trans-*isomerase. Longipin MIC in the high μM range compared to other AMPs, suggests that further experiments could be conducted to verify the influence of the Pro N-terminal peptide bond conformation on its activity.

## 5 Conclusions

Longipin is the first antimicrobial peptide isolated from an Opiliones order animal. It is constitutively expressed in the hemolymph, as observed for several other AMPs from arachnids, contributing to a wider understanding of the immunological system from different groups of animals.

Due to the appearance of pathogenic microorganisms resistant to modern antibiotics, the development of new antimicrobial drugs has become important. In this sense, the selectivity of longipin against microbial cells (yeast and bacteria) could be further explored to evaluate its molecular mechanisms and improve its antimicrobial activity.

This work also contributes to the characterization of a novel amyloid antimicrobial peptide and reinforces the relationship between amyloid pathogenesis and the mode of action of membrane active antimicrobial peptides.

## Supporting Information

S1 File LC-ESI-MS analysis of the fragments obtained after acetylation and enzymatic digestion (endoprotease Glu-C) of the peptide from the P5a fraction. (a) Ion chromatogram of the analysis, where most intense peaks are marked in blue (35 min) and green (40 min) traces. The digestion fragments ions eluted in the blue and green interval of time are shown in the panel b and c, respectively **(Figure A). “De novo” sequencing of longipin fragments obtained after the acetylation and enzymatic digestion (endoprotease Glu-C) of the P5a fraction.** The CID spectra from (a) N-terminal fragment 2+ ion ([M+2H]^2+^, *m/z* 467.59) and (b) C-terminal fragment 2+ ion ([M+2H]^2+^, *m/z* 710.8) acquired under a 15 V potential in the collision cell. The -*y* and -*b* fragments are marked on the top of the figure in red and blue, respectively. Acetylated lysine residues (K^AC^) identified in the primary sequence allowed the differentiation between Lys and Gln. The presence of immonium ion from K^AC^ with ammonia neutral loss (K^AC^-NH_3_) in the low range *m/z* (126) confirmed the chemical modification of this residue **(Figure B)**. **Evaluation of synthetic longipin homogeneity.** (a) RP-HPLC profile of the purified synthetic longipin obtained with an analytical column Shim-pak VP-ODS (5 μM, 4.6 × 250 mm) in a linear gradient from 15% to 40% of ACN/TFA 0.05% in H_2_O/TFA 0.05% during 28 min at 1.0 ml/min flow rate. The base line of the profile was set in 0 mAbs for integration of peaks. (b) ESI-MS analysis of the major peak (area = 132) showing expected average mass of synthetic longipin (2127.5 Da) after *m/z* values deconvolution. Ions related to peptide charged with K^+^ adduct (2166.6 Da) were also detected in the spectrum **(Figure C). Comparison between native (blue) and synthetic (green) longipin CID spectra (Figure D). Effect of longipin on the viability of VERO cells.** After 24 h treatment with longipin at different concentrations, cells viability was evaluated by MTT method **(Figure E). Amide I’ band from deconvoluted FT-IR spectra of longipin in solution (D**_**2**_**O) and in the presence of MLV composed by POPC or POPG:POPC (1:1 molar ratio).** The dashed lines are second derivative used for band fitting **(Figure F). Longipin concentration-dependent dye leakage from POPG:POPC vesicles loaded with CF 80 mM.** The assay was performed under low ionic strength conditions NaCl 10 mM/sucrose 140 mM with longipin at increasing concentrations (1, 5, 10 or 20 μM) added in ~ 200 s. CF fluorescence was monitored (λ_EX_ = 474 nm / λ_EM_ = 518 nm), and Triton X-100 was added to a final 0.1% concentration to achieve the maximum fluorescence intensity **(Figure G). MIC values of streptomycin against microorganisms evaluated under microbial growth inhibition assay (Table A).**
(PDF)
