# Supplementary material for: Longipin: An Amyloid Antimicrobial Peptide from the Harvestman Acutisoma longipes (Arachnida: Opiliones) with Preferential Affinity for Anionic Vesicles
Source: PLoS One. 2016 Dec 20;11(12):e0167953. doi: 10.1371/journal.pone.0167953 (PMC5172563; doi:10.1371/journal.pone.0167953)
Supplement: S1 File — (a) Ion chromatogram of the analysis, where most intense peaks are marked in blue (35 min) and green (40 min) traces. The digestion fragments ions eluted in the blue and green interval of time are shown in the panel b and c, respectively (Figure A). “De novo” sequencing of longipin fragments obtained after the acetylation and enzymatic digestion (endoprotease Glu-C) of the P5a fraction. The CID spectra from (a) N-terminal fragment 2+ ion ([M+2H]2+, m/z 467.59) and (b) C-terminal fragment 2+ ion ([M+2H]2+, m/z 710.8) acquired under a 15 V potential in the collision cell. The -y and -b fragments are marked on the top of the figure in red and blue, respectively. Acetylated lysine residues (KAC) identified in the primary sequence allowed the differentiation between Lys and Gln. The presence of immonium ion from KAC with ammonia neutral loss (KAC-NH3) in the low range m/z (126) confirmed the chemical modification of this residue (Figure B). Evaluation of synthetic longipin homogeneity. (a) RP-HPLC profile of the purified synthetic longipin obtained with an analytical column Shim-pak VP-ODS (5 μM, 4.6 × 250 mm) in a linear gradient from 15% to 40% of ACN/TFA 0.05% in H2O/TFA 0.05% during 28 min at 1.0 ml/min flow rate. The base line of the profile was set in 0 mAbs for integration of peaks. (b) ESI-MS analysis of the major peak (area = 132) showing expected average mass of synthetic longipin (2127.5 Da) after m/z values deconvolution. Ions related to peptide charged with K+ adduct (2166.6 Da) were also detected in the spectrum (Figure C). Comparison between native (blue) and synthetic (green) longipin CID spectra (Figure D). Effect of longipin on the viability of VERO cells. After 24 h treatment with longipin at different concentrations, cells viability was evaluated by MTT method (Figure E). Amide I’ band from deconvoluted FT-IR spectra of longipin in solution (D2O) and in the presence of MLV composed by POPC or POPG:POPC (1:1 molar ratio). The dashed lines are second de [file pone.0167953.s001.pdf]

Supplementary Material

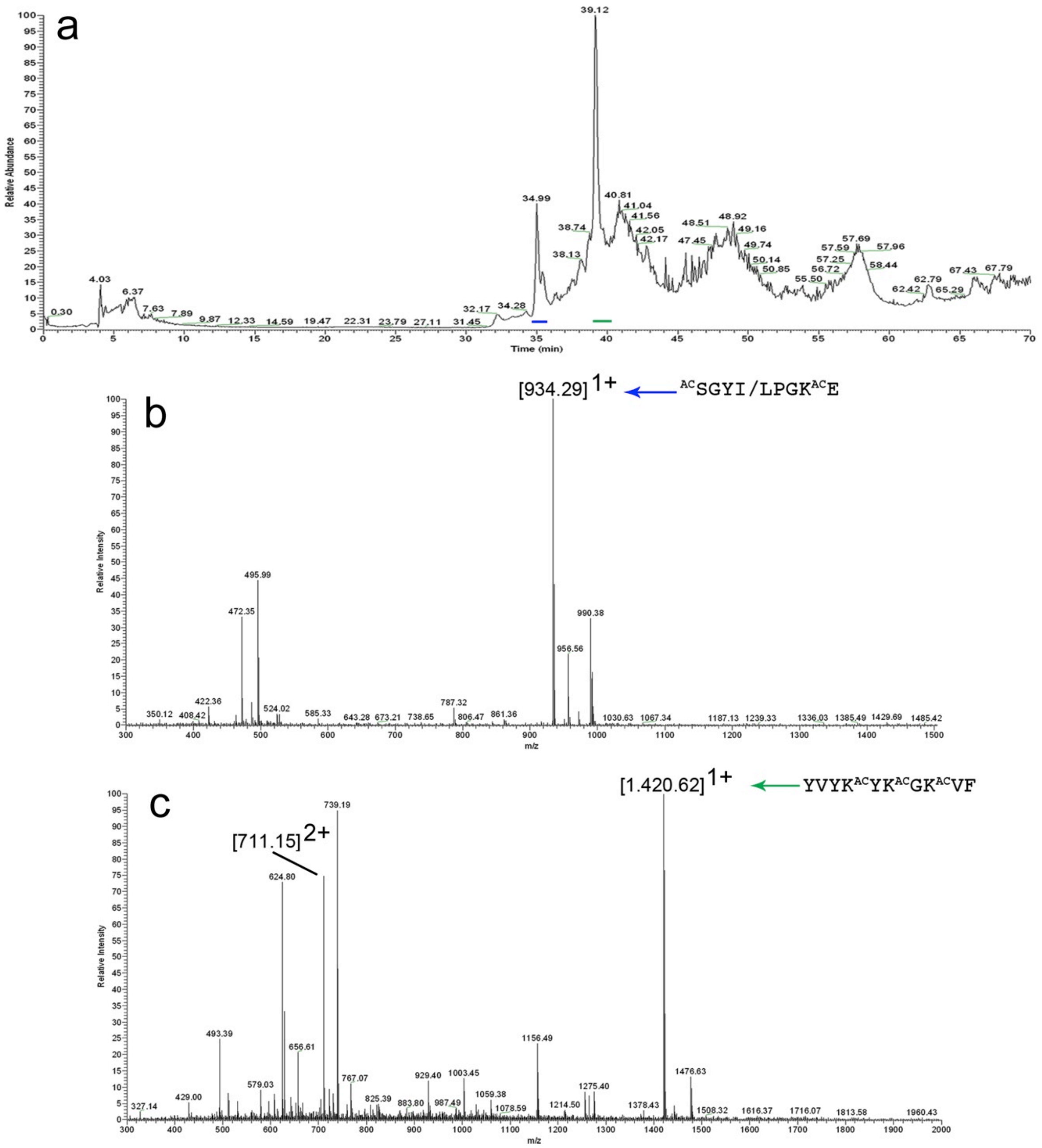

Figure A. LC-ESI-MS analysis of the fragments obtained after acetylation and enzymatic digestion (endoprotease Glu-C) of the peptide from the P5a fraction. (**a**) Ion chromatogram of the analysis, where most intense peaks are marked in blue (35 min) and green (40 min) traces. The digestion fragments ions eluted in the blue and green interval of time are shown in the panel **b** and **c**, respectively.

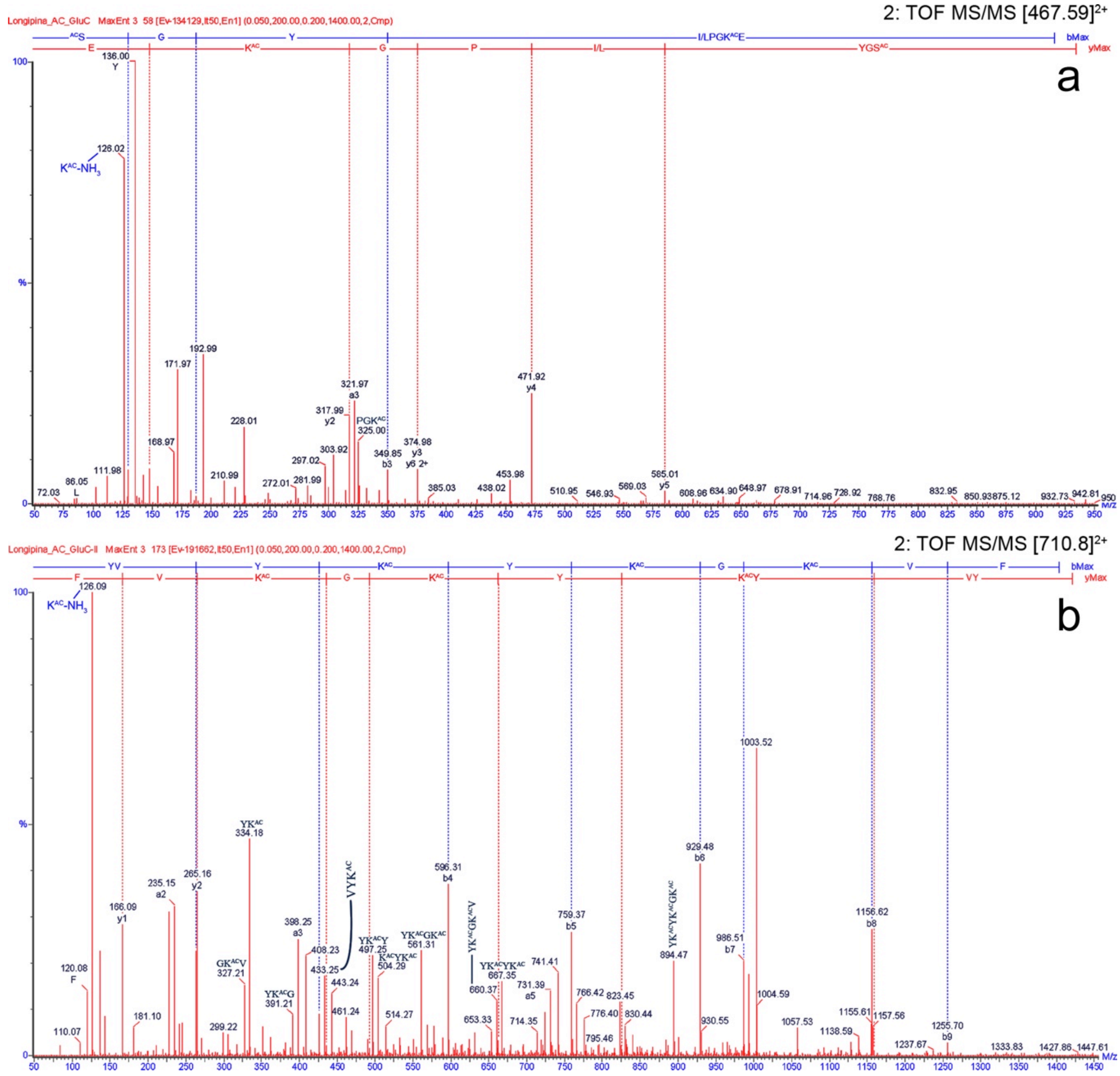

Figure B. “De novo” sequencing of longipin fragments obtained after the acetylation and enzymatic digestion (endoprotease Glu-C) of the P5a fraction. The CID spectra from (**a**) N-terminal fragment 2+ ion ($[M+2H]^{2+}$, *m/z* 467.59) and (**b**) C-terminal fragment 2+ ion ($[M+2H]^{2+}$, *m/z* 710.8) acquired under a 15 V potential in the collision cell. The *-y* and *-b* fragments are marked on the top of the figure in red and blue, respectively. Acetylated lysine residues (K^{AC}) identified in the primary sequence allowed the differentiation between Lys and Gln. The presence of immonium ion from K^{AC} with ammonia neutral loss (K^{AC}-NH_3) in the low range *m/z* (126) confirmed the chemical modification of this residue.

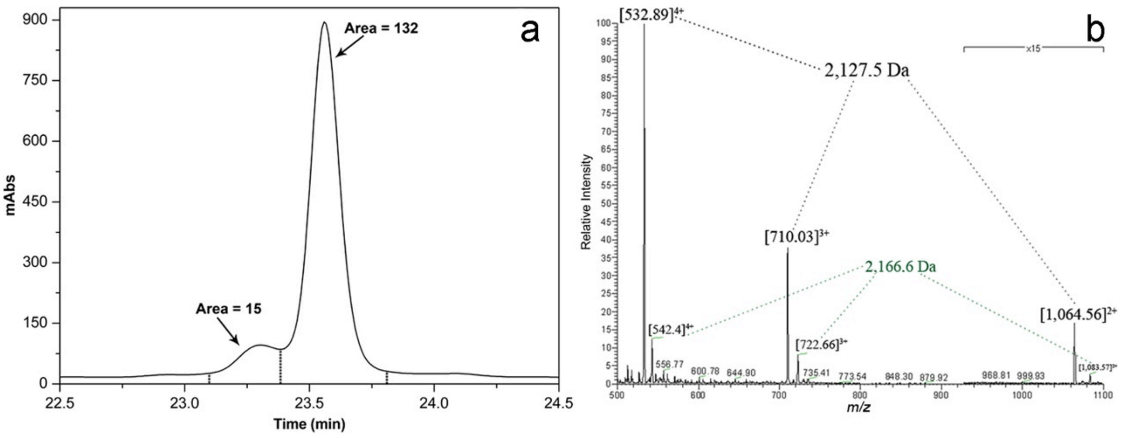

Figure C. Evaluation of synthetic longipin homogeneity. (**a**) RP-HPLC profile of the purified synthetic longipin obtained with an analytical column Shim-pak VP-ODS (5 μM, 4.6 × 250 mm) in a linear gradient from 15% to 40% of ACN/TFA 0.05% in H_2O/TFA 0.05% during 28 min at 1.0 ml/min flow rate. The base line of the profile was set in 0 mAbs for integration of peaks. (**b**) ESI-MS analysis of the major peak (area = 132) showing expected average mass of synthetic longipin (2127.5 Da) after *m/z* values deconvolution. Ions related to peptide charged with K^+ adduct (2166.6 Da) were also detected in the spectrum.

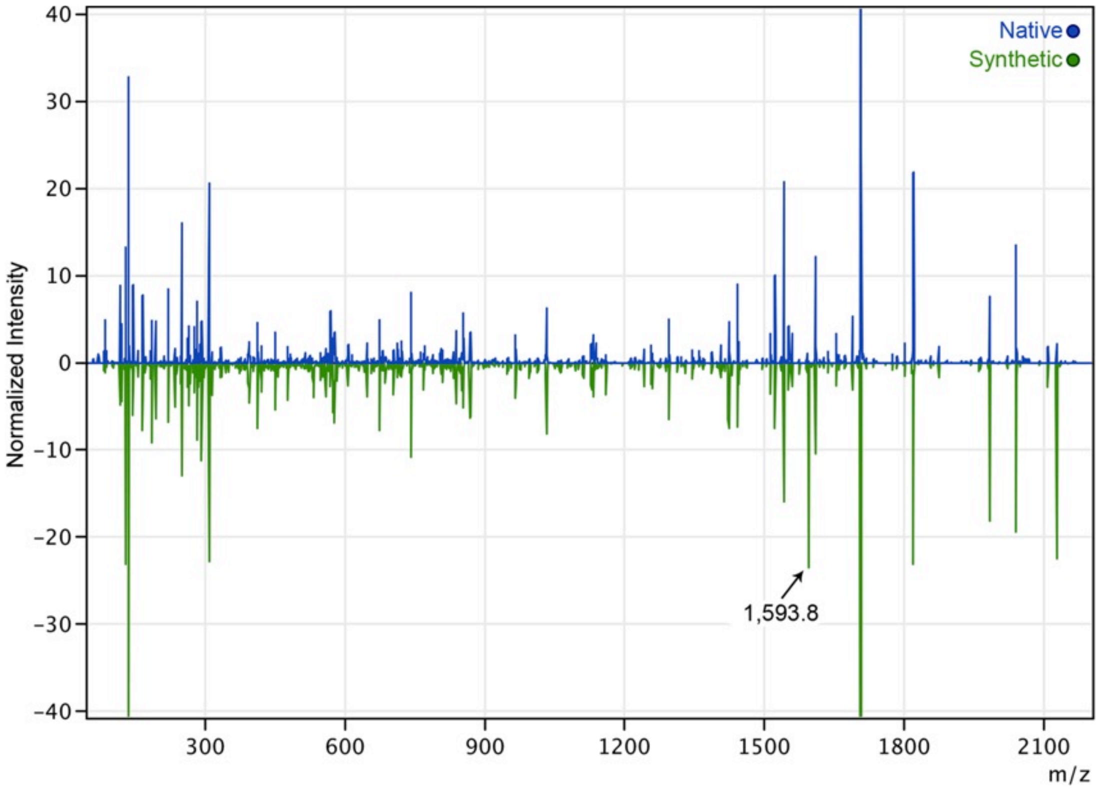

Figure D. Comparison between native (blue) and synthetic (green) longipin CID spectra.

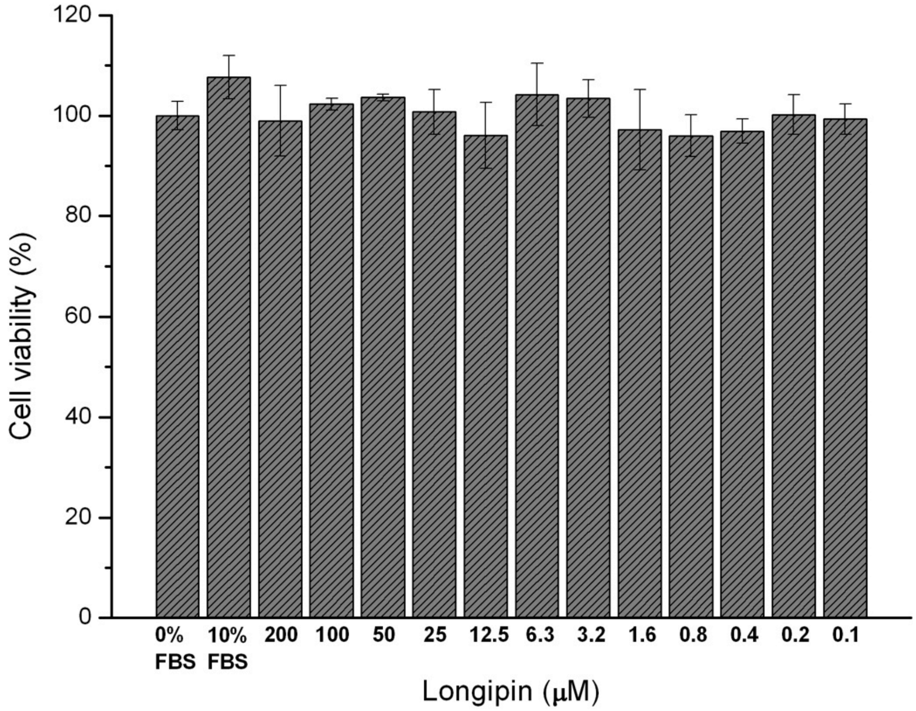

Figure E. Effect of longipin on the viability of VERO cells. After 24 h treatment with longipin at different concentrations, cells viability was evaluated by MTT method.

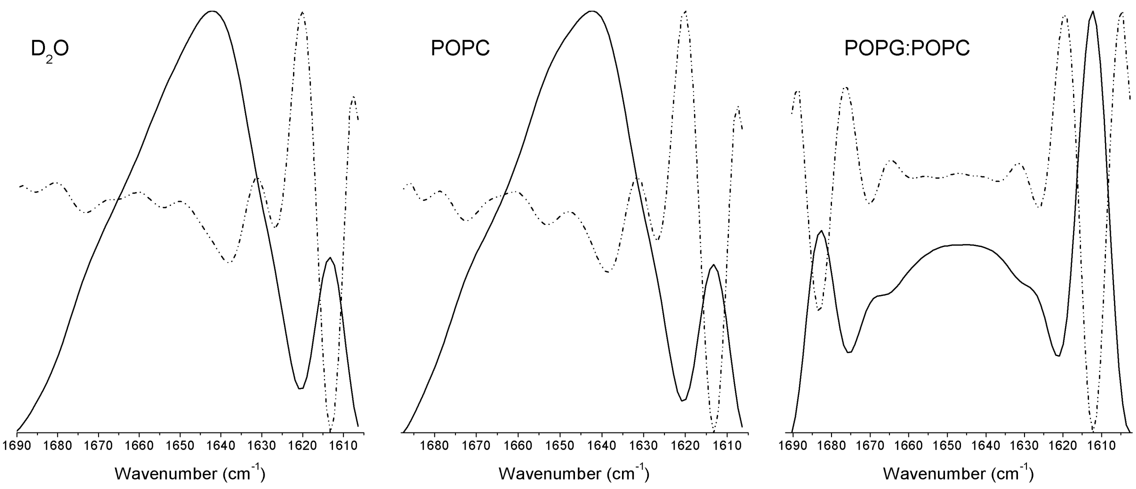

Figure F. Amide I' band from deconvoluted FT-IR spectra of longipin in solution (D_2O) and in the presence of MLV composed by POPC or POPG:POPC (1:1 molar ratio). The dashed lines are second derivative used for band fitting.

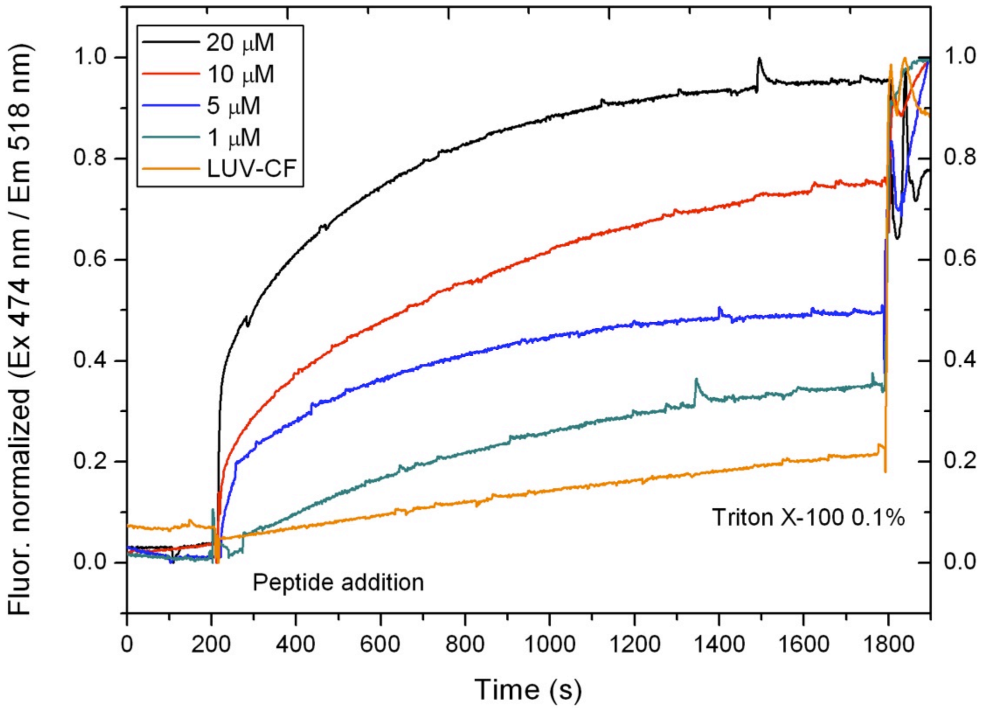

Figure G. Longipin concentration-dependent dye leakage from POPG:POPC vesicles loaded with CF 80 mM. The assay was performed under low ionic strength conditions NaCl 10 mM/sucrose 140 mM with longipin at increasing concentrations (1, 5, 10 or 20 μM) added in ~ 200 s. CF fluorescence was monitored (λ_{EX} = 474 nm / λ_{EM} = 518 nm), and Triton X-100 was added to a final 0.1% concentration to achieve the maximum fluorescence intensity.

Table A. MIC values of streptomycin against microorganisms evaluated under microbial growth inhibition assay.

Microorganisms	MIC (µg/ml)
**- Gram-positive bacteria**	
Staphylococcus aureus ATCC29213	NE
Staphylococcus epidermidis ATCC12228	NE
Micrococcus luteus BR2	NE
Micrococcus luteus A270	NE
**- Gram-negative bacteria**	
Pseudomonas aeruginosa ATCC27853	0.062 – 0.125
Escherichia coli D31	0.015 – 0.03
Escherichia coli SBS363	NE
Serratia marcescens ATCC4112	0.062 – 0.125
Enterobacter cloacae β12	NE
**- yeasts**	
Candida albicans MDM8	250 – 500
Candida albicans IOC4558	125 – 250
Candida tropicalis IOC4560	125 – 250
Candida guilliermondii IOC4557	0.25 – 0.5
**- filamentous fungi**	
Aspergillus niger	250 – 500
Cladosporium herbarum ATCC26362	500 – 1000
Paecilomyces farinosus IBCB251	1000 – 2000

NE – not evaluated (complete growth inhibition was observed with streptomycin 2000 µg/ml).
